# A case of delirium following treatment with low dose mirtazapine and pregabalin

**DOI:** 10.1192/j.eurpsy.2024.1448

**Published:** 2024-08-27

**Authors:** T. A. Manolis, D. Argyropoulos, N. Kokras, A. M. Politis

**Affiliations:** First Department of Psychiatry, National and Kapodistrian University of Athens, Athens, Greece

## Abstract

**Introduction:**

Pregabalin is a gamma-aminobutyric acid analogue used for the treatment of neuropathic pain, partial-onset-seizures, fibromyalgia, and anxiety disorders. Mirtazapine is an atypical antidepressant used in major depression and often prescribed off-label for insomnia. Delirium, an acute confusional state, is a very rare adverse reaction of both medications.

**Objectives:**

We report a case of an elderly patient treated with low dose pregabalin and mirtazapine who developed drug-induced delirium which resolved rapidly upon withdrawal of both drugs

**Methods:**

A 75-year-old woman was admitted for symptoms of anxiety, various bodily complaints (dysphagia, headache, tinnitus, weakness) and sleep-onset insomnia over the preceding 2 months. On admission, examination revealed an apparently anxious, uneasy and emotional looking patient. Mini mental state examination, as well as clock drawing and copying were normal, suggesting absence of cognitive impairment. Physical examination was unrevealing except for high blood pressure recordings (150/90 mmHg). Laboratory testing indicated creatinine at 1.19 mg/dl, with a creatinine clearance moderately decreased at 38 ml/min. Upon admission, she was placed on pregabalin 25 mg bid and mirtazapine 30 mg ¼ tablet qd.

**Results:**

Three days after admission, pregabalin was increased to 25 mg tid. On the same day and about 2 hours after the night dose, the patient acutely developed delirium: she presented confusion, disorientation, incoherence, restlessness and deterioration of her anxiety. On physical examination she was afebrile with no hypertonia or ataxia. An urgent brain magnetic resonance imaging was grossly unrevealing. Pregabalin and mirtazapine were discontinued, as a drug-induced delirium was suspected. She received as a symptomatic treatment lorazepam progressively up to 4 mg qd. Symptoms of delirium resolved rapidly, and she was discharged days later with full functional recovery

**Conclusions:**

Cases of delirium have been described following treatment with pregabalin, but in significantly higher doses. Pregabalin relies heavily on renal clearance for its excretion and the dose should be adjusted in patients with creatine clearance below 60 ml/min. As our patient had a moderate decrease in renal clearance, we prescribed a dose within suggested limits, but in combination with mirtazapine led to the appearance of a drug-induced delirium. In conclusion, combined therapy with low-dose pregabalin and mirtazapine seems to account for the development of delirium in our patient as based on its temporal association with the initiation of this drug combination and its prompt resolution upon withdrawal of these two agents

**Disclosure of Interest:**

None Declared

